# An experimental study of tuned liquid column damper controlled multi-degree of freedom structure subject to harmonic and seismic excitations

**DOI:** 10.1371/journal.pone.0269910

**Published:** 2022-06-13

**Authors:** Mati Ullah Shah, Muhammad Usman

**Affiliations:** School of Civil and Environmental Engineering (SCEE), National University of Sciences and Technology (NUST), Islamabad, Pakistan; Fuzhou University, CHINA

## Abstract

A tuned liquid column damper (TLCD) is a passive vibration control device that not only mitigates unwanted structural vibrations but also acts as a water storage facility in a building. These aspects of TLCD make its application specifically suited for building structures. Previously, many experimental works on TLCDs have been conducted considering a single degree of freedom (SDOF) structure. However, the performance of TLCDs to control the response of multi-degree of freedom (MDOF) structure has rarely been studied experimentally. Therefore, this study has investigated the performance of a tuned liquid column damper (TLCD) on a multi-degree of freedom (MDOF) structure using shake table testing. A four-storey steel frame structure equipped with TLCD at the top of the fourth storey has been studied. Experimental normalized frequency response curves for MDOF structure equipped with TLCD have been determined. For this purpose, a series of harmonic loadings including frequencies 0.65 Hz, 1.17 Hz, 1.30 Hz, 1.43 Hz and 1.95 Hz have been applied in addition to historic earthquake loading. Peak and root-mean-square (RMS) accelerations have been discussed in detail for all the applied loadings at each storey level of the structure. For comparison purposes, the percentage reductions in peak and RMS accelerations have been calculated and compared. Also, RMS displacements and inter-storey drifts have been presented for resonant and seismic excitations. Both in time and frequency domains, responses of controlled MDOF structure have been analyzed and compared with uncontrolled structure. Results confirmed that TLCD has improved the MDOF structure responses at harmonic loadings frequencies near resonance and historic earthquake excitations. Furthermore, the improvement in the responses of MDOF structure with TLCD is more prominent at harmonic loadings compared to historic earthquake loading.

## 1. Introduction

High-rise structures are vulnerable to structural vibrations induced by wind and earthquake loadings [1, 2]. These structural vibrations if not mitigated properly can cause discomfort to the occupants [3, 4] and possible structural failures [5–7]. Therefore, coping with these unwelcoming structural vibrations and keeping the structures within the serviceability limits are the key concerns of structural engineers [8, 9]. Traditionally structures have been designed such that the amplitude of the vibrations could be minimized [10–13]. However, vibration control systems have also been employed to mitigate these vibrations by enhancing energy dissipation [14–16]. These systems are mainly passive, active, semi-active, and hybrid [16–19]. Compared to active, semi-active and hybrid systems, passive damping systems are the most reliable and economical vibration mitigation systems [20, 21].

Passive vibration systems include tuned mass dampers (TMDs), tuned liquid dampers (TLDs), tuned liquid column dampers (TLCDs), combined tuned dampers (CTDs), etc. TMD consists of a mass attached to the host structure in the form of a pendulum. The frequency of the pendulum is tuned according to the host structure governing vibrational mode frequency [22], so it can effectively dissipate unwanted structural vibrations. While TLD consists of a container partially filled with a fluid preferably water. It was first introduced in the 1980s. It dissipates the energy through the sloshing effect of the liquid [21] and the sloshing of the liquid is tuned according to the host structure frequency for optimal results [23]. TLDs had been studied for controlling the structure response against wind [23–25] and seismic loadings [26, 27]. The complexity of non-linear modelling of TLD due to wave breaking phenomena of sloshing liquid inside container [28, 29] and damping enhancement elements of TLD [30] made it unfavourable. Therefore, the researchers extended the concept of TLDs to TLCDs. TLCD was first introduced by Sakai et al. in 1989 [31]. TLCD consists of a U shape [32] uniform cross-sectional area column filled with a fluid having an orifice in the horizontal section of the tube. It dissipates structural vibration energies through head loss of the fluid while it passes through the orifice [22]. TLCDs have many advantages over earlier TLDs [33] like (i) the damping ability of TLCDs is flexible and can be controlled by adjusting orifice opening, (ii) the frequency of TLCD can be tuned easily by adjusting the length of the liquid column, (iii) the geometry of TLCD can be varied as per design requirements of the structure. These advantages make TLCDs more compatible and applicable to real structures [33–35].

Keeping in view the ability of TLCDs in terms of applications to practical structures, numerous efforts have been made to verify the effectiveness of TLCDs for controlling structural vibrations induced by wind and seismic loadings since 1989 [22, 33]. Numerical and experimental studies have been conducted to derive optimal design parameters for TLCDs [36–38]. Researchers have improved the potency of TLCDs using optimal tuning ratios [39], mass ratios [40], variable orifice opening [41], denser fluids [42, 43], the variable cross-section area of the tube [32, 44–46], and modified shapes [44, 47–50]. Even Xue et al. have demonstrated that TLCDs can be as effective as TMDs against wind loadings if design parameters have been properly selected [35]. Shum has suggested optimal design parameters for TLCDs for reducing wind-induced vibrations [39]. With time, various modified versions of TLCDs have been introduced and studied for better performance and efficiency [15, 20, 33, 51–53]. But those modifications in TLCD have also brought complexity to the system. For example, the traditional TLCD is modelled as a single degree of freedom (SDOF). However, in the modified versions degrees of freedom associated with the damper are increased. Like in a tuned liquid column ball damper (TLCBD), two degrees of freedom are associated with the damper. Apart from the analytical complexities of the modified systems, the practical implementation is also a hurdle. One of the other disadvantages of TLCBD is that it is only good if the structure is vibrating at low frequencies [51]. From a practical application point of view, Sakai’s TLCD is the simplest and owes considerable advantages including low cost, easy installation and handling process, and very low maintenance cost [31, 33, 34].

Several studies have been conducted to establish optimal design guidelines for TLCDs against wind [40] and seismic excitations [54]. In literature, the design guidelines and optimal design parameters have been developed for TLCDs considering a single degree of freedom (SDOF) host structure [38–40]. Also, most of the experimental studies on TLCDs have been performed using SDOF structures. Some analytical studies on TLCDs have been conducted for multi-degree of freedom structures (MDOF) [55–58]. However, experimental studies on TLCDs to verify their effectiveness for controlling MDOF structure responses are very rare. Tanveer et al. studied TLCD and TLCBD for MDOF structures [59]. However, in that study structure was tested against low-frequency loadings [51] compared to its fundamental frequency, where TLCBD was more effective than TLCD. Therefore, progressive efforts are required to explore the performance of TLCDs on MDOF structures. There is a need to do extensive experimental work on TLCDs for improving MDOF structure responses. Because these efforts will open a door to implementing TLCDs in practical buildings.

In the present study, an attempt is made to experimentally study the effectiveness of TLCD for MDOF structure using shake table testing which is previously very rare to find. Total two experimental setups are studied under harmonic and seismic loadings. These setups include uncontrolled MDOF structure and MDOF structure equipped with TLCD. In the current work, normalized frequency responses are experimentally developed for MDOF structure which has never been done previously. Another, uniqueness of this work is that the structure response has been observed experimentally at five different harmonic frequency loadings that lie in three different regions, (i) before the resonant region, (ii) near-resonant region, and (iii) post resonant region. The MDOF structure used is a four-storey steel frame structure that has constant storey mass, height, and stiffness. Time history responses of both setups have been drawn and compared against resonant and seismic loadings. The root-mean-square (RMS) and peak accelerations values for each storey of both the setups have been discussed in detail and for comparison percentage reductions in the responses have been determined. RMS displacements and drift responses have been also analyzed. For a better understanding of the structure, responses have also been analyzed in the frequency domain.

## 2. Analytical background

**Fig 1:**
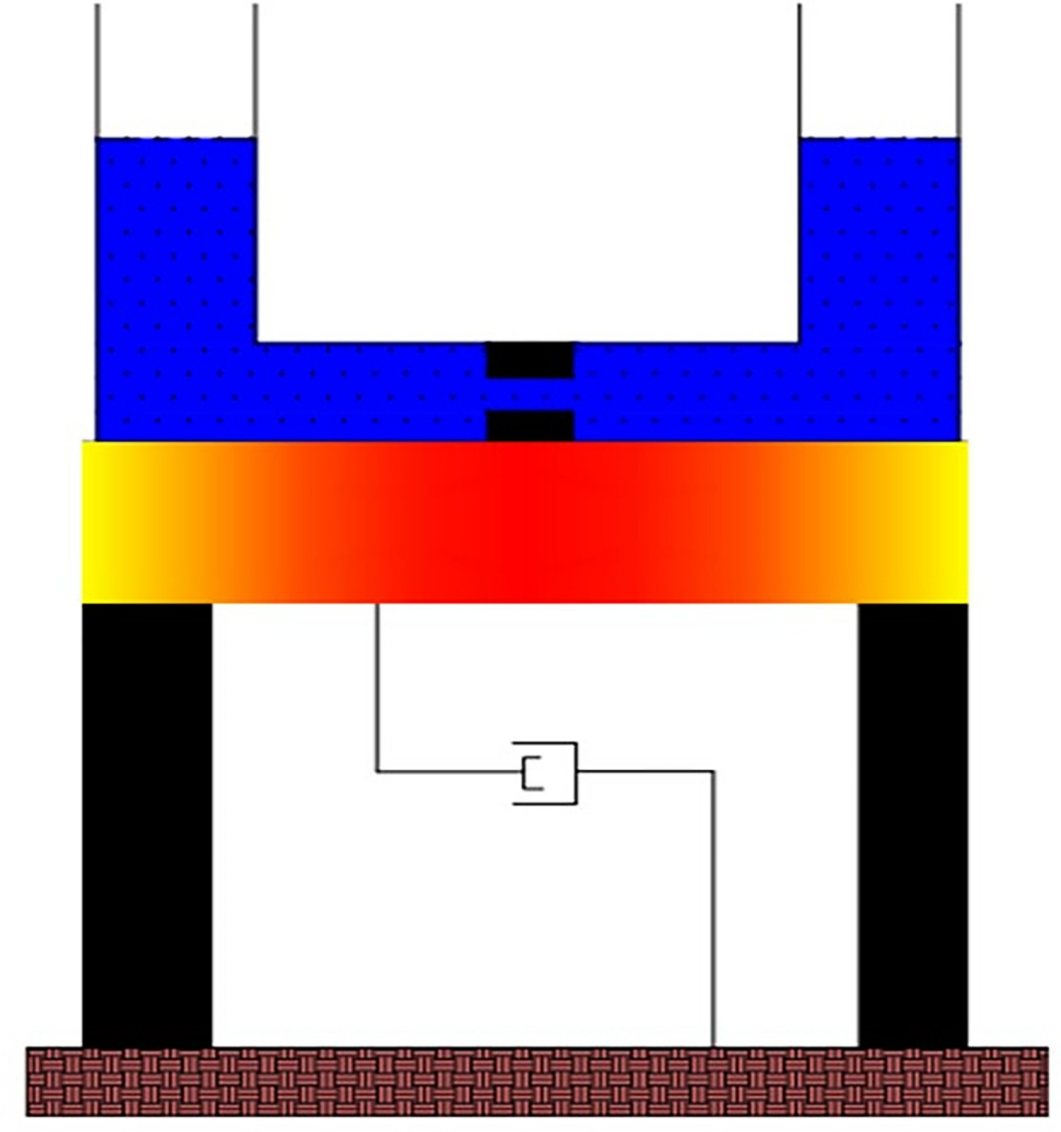
Schematic view of Tuned Liquid Column Damper (TLCD) on an SDOF system.

TLCD consists of U shape column filled with a fluid having an orifice at the center of the horizontal section of the tube as shown in Fig 1. The natural frequency of the TLCD depends on the total length of the liquid column and its damping capacity can be controlled by adjusting the orifice opening. TLCD dissipates vibrations energies through head loss of the fluid while it is flowing throw the orifice. The TLCD is an SDOF system. The governing equation of motion for the liquid part of TLCD was derived by Sakai et al. [31] as follows.


$$m_{f}\ddot{y}+\frac{1}{2}\rho A_{o}\sigma |\dot{y}|\dot{y}+2\rho gAy+\alpha m_{f}{\ddot{x}}_{s}=0$$ (1)

The coupled governing equation of motion for the structure equipped with TLCD [31] follows as.


$$(m_{s}+m_{f}){\ddot{x}}_{s}+c_{s}{\dot{x}}_{s}+k_{s}x_{s}+\alpha m_{f}\ddot{y}=f_{ext(t)}$$ (2)

In the above equations *m*_*f*_ is the mass of the fluid, *A*_*o*_ is the area of the orifice, *σ* is the co-efficient of head loss, *y* is the displacement of the fluid surface, *ρ* is the density of the fluid, *g* is the gravitational acceleration, *A* is the cross-section area of the tube, *α* is the length ratio (the horizontal length of the liquid total length of the fluid), *m*_*s*_, *x*_*s*_, *c*_*s*_, and *k*_*s*_ are the mass, displacement, damping, and stiffness of the structure equipped with TLCD, and *f*_*ext*(*t*)_ is the external loading. The single and double dots in the superscript of variables present velocity and displacement respectively. Equipping MDOF structure with TLCD, the structure parameters associated with the degree of the freedom of MDOF structure on which TLCD is placed should be considered in Eq (2). Like an MDOF structure equipped with TLCD at the top of its fourth storey. Then in Eq (2), *m*_*s*_, *k*_*s*_ and *c*_*s*_ will be the mass, stiffness, and damping of the fourth storey respectively. The generalized matrix form for governing equations of motion for n^th^ MDOF structures can be expressed as:

$${\left[M\right]}_{n+1*n+1}\{{\ddot{x}}_{s}\}+{\left[C\right]}_{n+1*n+1}\dot{{\{x}_{s}\}}+{\left[K\right]}_{n+1*n+1}\{x_{s}\}=-{\left[M\right]}_{n+1*n+1}\{z\}{\ddot{x}}_{g}$$ (3)

In the above Eq (3), [M], [C], and [K] are the mass, stiffness and damping matrix respectively having order n+1 * n+1, where n represents the degree of freedom of the host structure and 1 is for the degree of freedom of TLCD. $\left\{{\ddot{x}}_{s}\right\}$, $\dot{{\{x}_{s}\}}$, {*x*_*s*_}, and {*z*} are the acceleration, velocity, displacement, and influences co-efficient vectors respectively.

## 3. Experimental investigation

### 3.1. Test specimen

**Fig 2:**
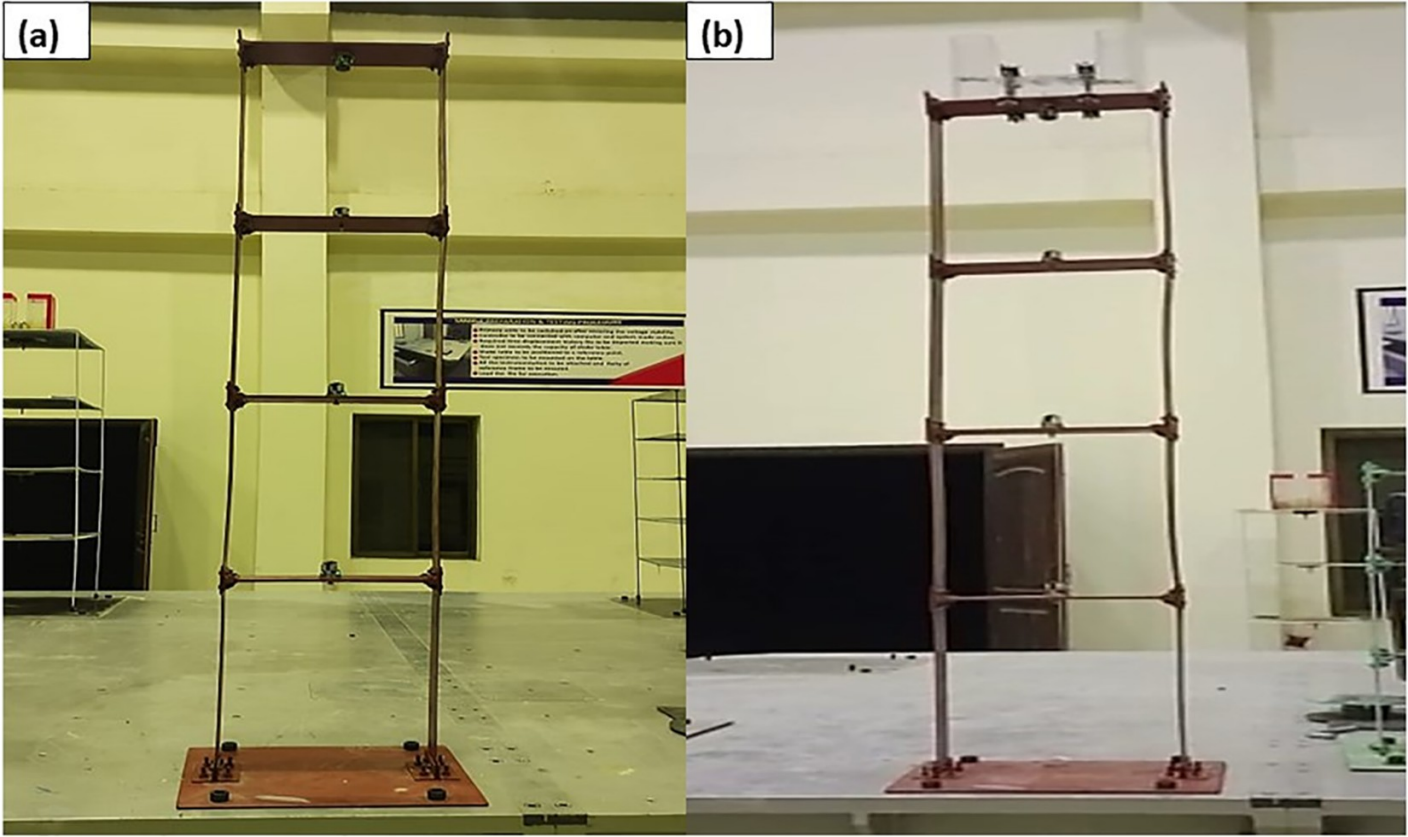
Frame structures (a) Uncontrolled MDOF structure, (b) MDOF structure equipped with TLCD.

**Fig 3:**
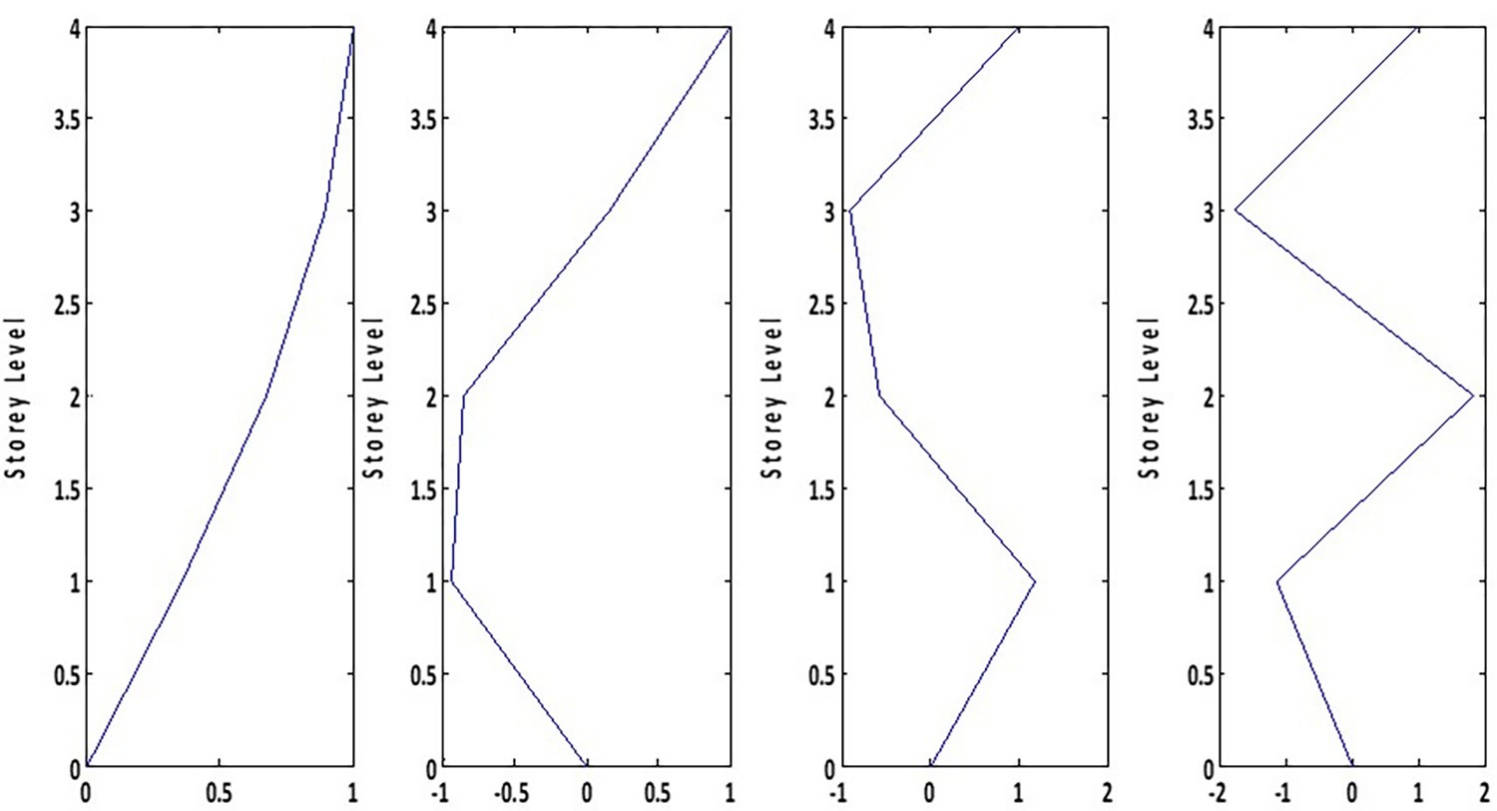
Mode shapes of the structure.

For the experimental study, a four-storey steel frame structure has been used. The frame has a constant storey height (0.508 m), width (0.381 m) and mass (4.825 kg) as shown in Fig 2. The frame structure has been scaled according to Inamdar et al. [60]. The length, width, and thickness of the column plate of the frame are 508 mm, 100 mm, and 2 mm respectively. While the beam plate length, width, and thickness are 380 mm, 100 mm, and 8 mm respectively. The frame structure has been assembled by bolted connections. The modal properties of the uncontrolled and controlled frame structures have been analytically calculated using mass and stiffness matrixes as shown in Tables 1 and 2 respectively. The analytically derived mode shapes of the structure are shown in Fig 3. The TLCD design parameters used for the MDOF structure have been adopted from the literature [39, 40] as shown in Table 3. The following steps have been followed to design TLCD for the MDOF structure.

1. First analytically calculated the MDOF structure fundamental frequency.
2. Selected the tunning frequency ratio (0.98) based on the first governing fundamental vibrational mode.
3. For optimum results, a 5% mass ratio has been chosen for the liquid component of the TLCD.
4. The total length of the TLCD has been calculated from the frequency of TLCD.
5. The diameter of the tube has been calculated based on the length of TLCD and the volume of the fluid.
6. The length ratio and orifice blocking ratio have been selected as 0.8 and 0.5 respectively.

**Table 1:** Modal properties of steel frame used in experiments.

Modes	Natural Frequency (rad/sec)	Natural Frequency (Hz)	Time period (sec)	Modal participation factor	Storey Level	Lumped Mass (Kg)	Stiffness (N/m)	Damping (N-sec/m)
First	8.18	1.30	0.77	1.25	1	4.825	2480	1.720
Second	23.39	3.72	0.27	-0.36	2	4.825	2480	1.720
Third	34.50	5.49	0.18	0.14	3	4.825	2480	1.720
Fourth	42.86	6.82	0.15	-0.04	4	3.994	2480	1.720

**Table 2:** Modal properties of steel frame equipped with TLCD.

Modes	Natural Frequency (rad/sec)	Natural Frequency (Hz)	Time period (sec)
First	6.96	1.11	0.90
Second	9.16	1.46	0.68
Third	23.04	3.67	0.27
Fourth	35.01	5.57	0.17
Fifth	42.70	6.80	0.14

**Table 3:** Design parameters of TLCD.

Parameter	Values
Mass ratio, μ = *m*_*f*_/*m*_*s*_	0.05
Governing mode for TLCD design	First
Tuning frequency ratio	0.98
Frequency of TLCD (Hz)	1.27
Length ratio (*α*)	0.80
Orifice opening ratio	0.50
Fluid density (Kg/m^3^)	1000
Liquid Mass (gm)	965
The total length of liquid (cm)	30.53
The horizontal length of liquid (cm)	24.42
The vertical length of liquid (cm)	3.05

The shake table testing on MDOF structure with and without TLCD has been performed at the Structure Dynamic Lab of Military College of Engineering (MCE), Risalpur. The shake table used for testing is supplied by BESMAK, Turkey. It is a high capacity 4m x 4m servo-hydraulic unidirectional shake table. For acceleration data recording G-link-200 wireless accelerometers manufactured by Parker-LORD, NC, USA are used. The accelerometers are controlled through Sensor Connect for synchronizing and sampling data. For data acquisition and storage, the WSDA-2000-Wireless sensor data aggregator is used manufactured by Parker LORD, Cary, North Carolina, United States. MDOF structure is fixed on a 500 mm square base plate with a thickness of 12.5 mm. Four holes have been drilled at the corner of the base plate to fix the MDOF structure on the shake table.

### 3.2. Experimental procedure

**Fig 4:**
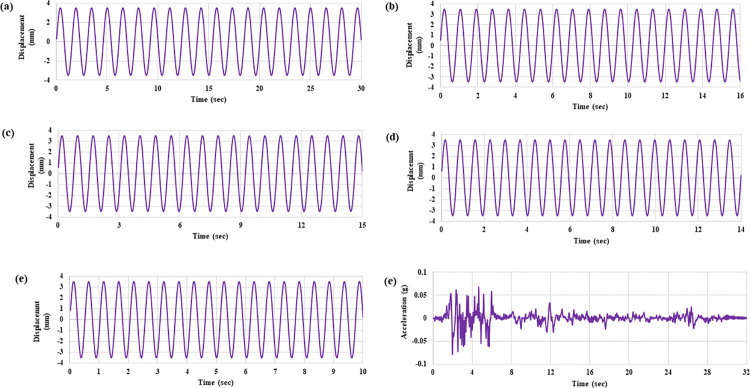
Applied loading acceleration time histories at: (a) 0.65 Hz, (b) 1.17 Hz, (c) 1.3 Hz, (d) 1.43 Hz, (e) 1.95 Hz, and (f) Seismic excitations.

**Fig 5:**
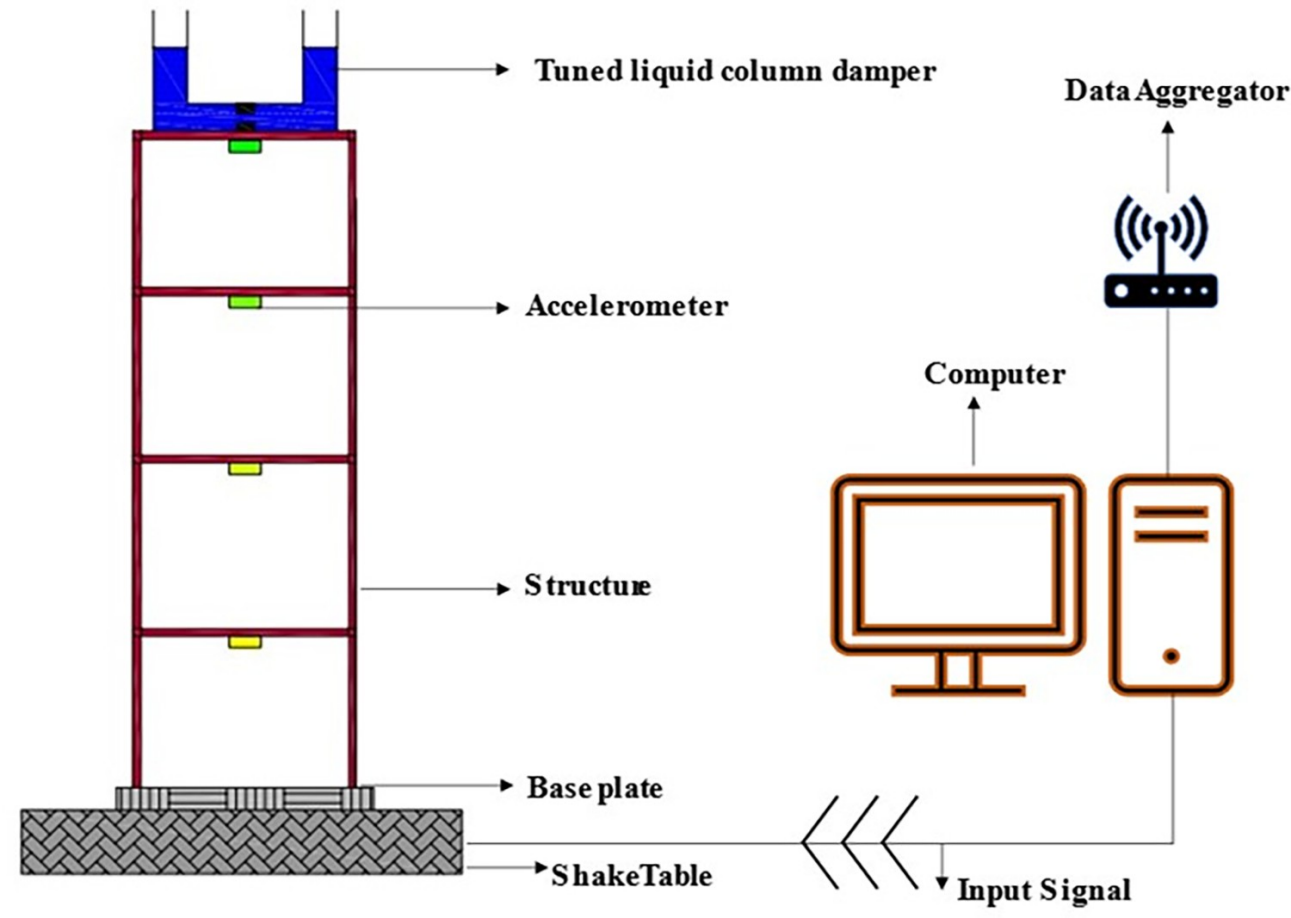
A schematic view of the experimental setup shows the MDOF structure equipped with TLCD.

In the present study, MDOF structure with and without TLCD is studied under harmonic and seismic loadings using a shake table. Five different harmonic loadings at constant amplitude (0.7 cm), fix the number of cycles (20), and varying frequencies (0.65 Hz, 1.17 Hz, 1.30 Hz, 1.43 Hz and 1.95 Hz) have been applied. The frequencies of harmonic loadings have been selected as 0.5 (0.65 Hz), 0.9 (1.17 Hz), 1 (1.30 Hz), 1.1 (1.43 Hz) and 1.5(1.95 Hz) times of the first mode frequency of the host structure. The reason behind selecting these frequencies is to capture the MDOF structure response in all three loading regions i.e., (i) before the resonant point (ii) at the resonant point and (iii) after the resonant point. For seismic response evaluation, 1940 EL Centro seismic excitations have been applied at a reduced scale. A total of five accelerometers one per storey of the structure and one on the floor of the shake stable were installed during shake table testing to record the acceleration responses. In a controlled setup, TLCD was placed at the top of the fourth storey of the structure in all the loading conditions. During testing the recorded data of accelerometers was transferred through WSDA-2000-Wireless sensor data aggregator in the computer system and stored in CSV file format. The recorded accelerometer data of both experimental arrangements have been processed in MATLAB. First, the recorded data is detrended and then processed to find the RMS and peak accelerations responses of each storey of the MDOF structure. Then detrended data has been processed to analyze and compare the acceleration time histories of both uncontrolled and controlled structures in the time and frequency domains. The time histories of the applied loadings are shown in Fig 4. While Fig 5 shows the schematic view of the whole experimental setup.

## 4. Results & discussions

### 4.1. Normalized frequency response

**Fig 6:**
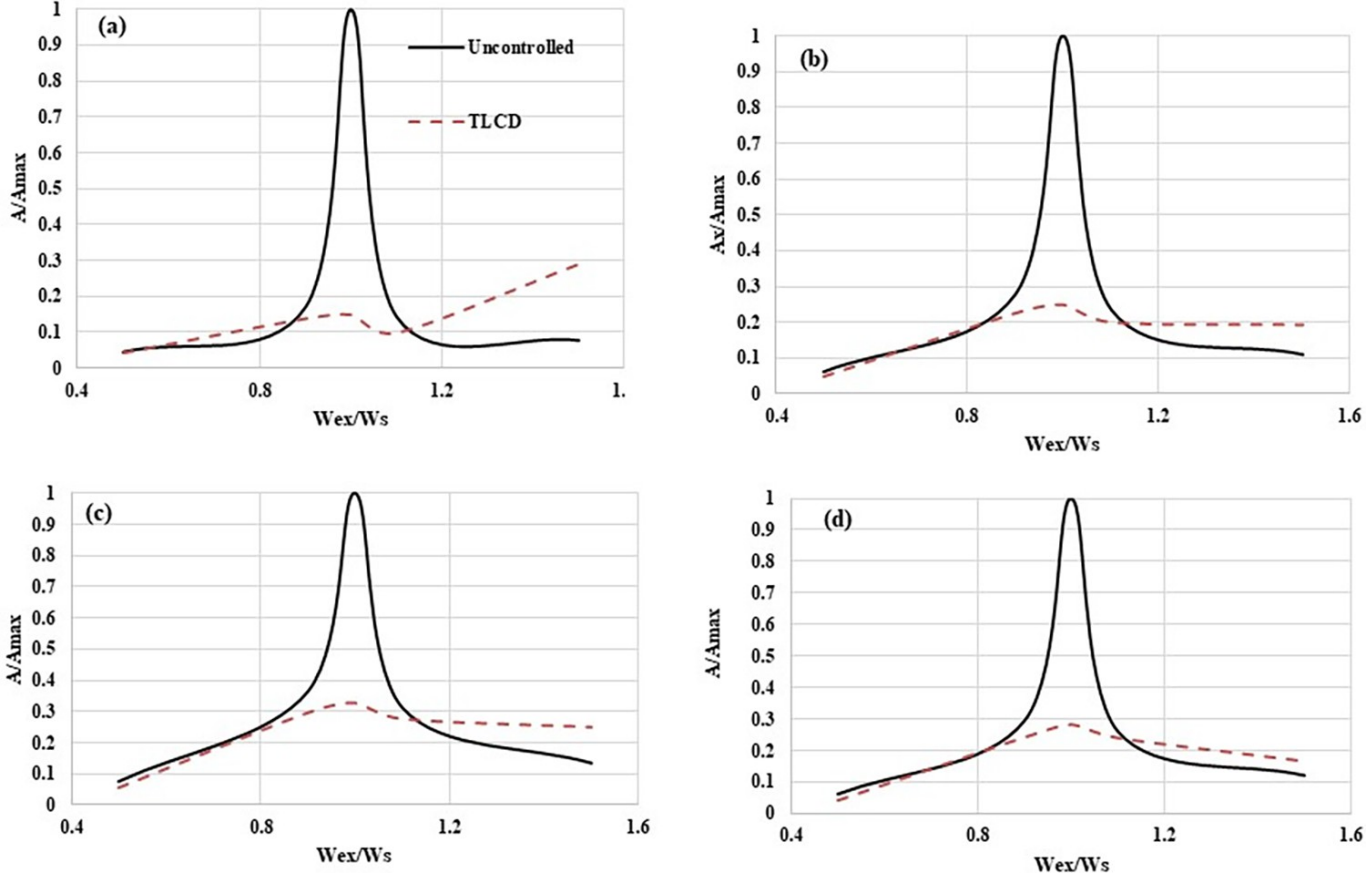
Normalized RMS acceleration responses of the MDOF structure equipped with and without TLCD, (a) Storey Level -01, (b) Storey Level -02, (c) Storey Level -03, (d) Storey Level -04.

Fig 6 exhibits the normalized frequency responses of the MDOF structure. Fig 6(A)–6(D) are the responses of the first, second, third, and fourth storey responses respectively. These normalized responses have been plotted by taking RMS accelerations values. On the x-axis, the (Wex/Ws) is the ratio of the applied harmonic loading frequency (Wex) to the frequency of the first fundamental mode (Ws) of the MDOF structure. At each storey level, the controlled structure response has been significantly reduced compared to the uncontrolled structure at the loadings frequencies ratios (Wex/Ws) lying in the range of 0.9 to 1.1. At a loading having a frequency ratio lower than 0.9, the response of structure at each storey is like the uncontrolled structure response. However, at a loading frequency ratio higher than 1.1 the response of a structure equipped with TLCD is slightly higher than the uncontrolled structure. The performance of TLCD in improving the MDOF structure near-resonant point is very prominent. The responses of the MDOF structure near the resonant point have been reduced by more than 85%, 75%, 70% and 75% compared to uncontrolled structure’s responses at storey-01, storey-02, storey-3, and storey-04 respectively. The response improvement of controlled structure compared to uncontrolled is because of better damping attributes of TLCD. During an excitation event, the TLCD attracts the vibrations from the host structure and dissipates them. But, the TLCD is effective for narrowband frequencies. Therefore, TLCD is designed mostly against the resonant frequency to protect the host structure from large amplitude vibration and possible failure. Also, in Fig 6, It can be seen that the TLCD is very effective in reducing the response of the structure in the resonance frequency range.

### 4.2. Acceleration response

**Fig 7:**
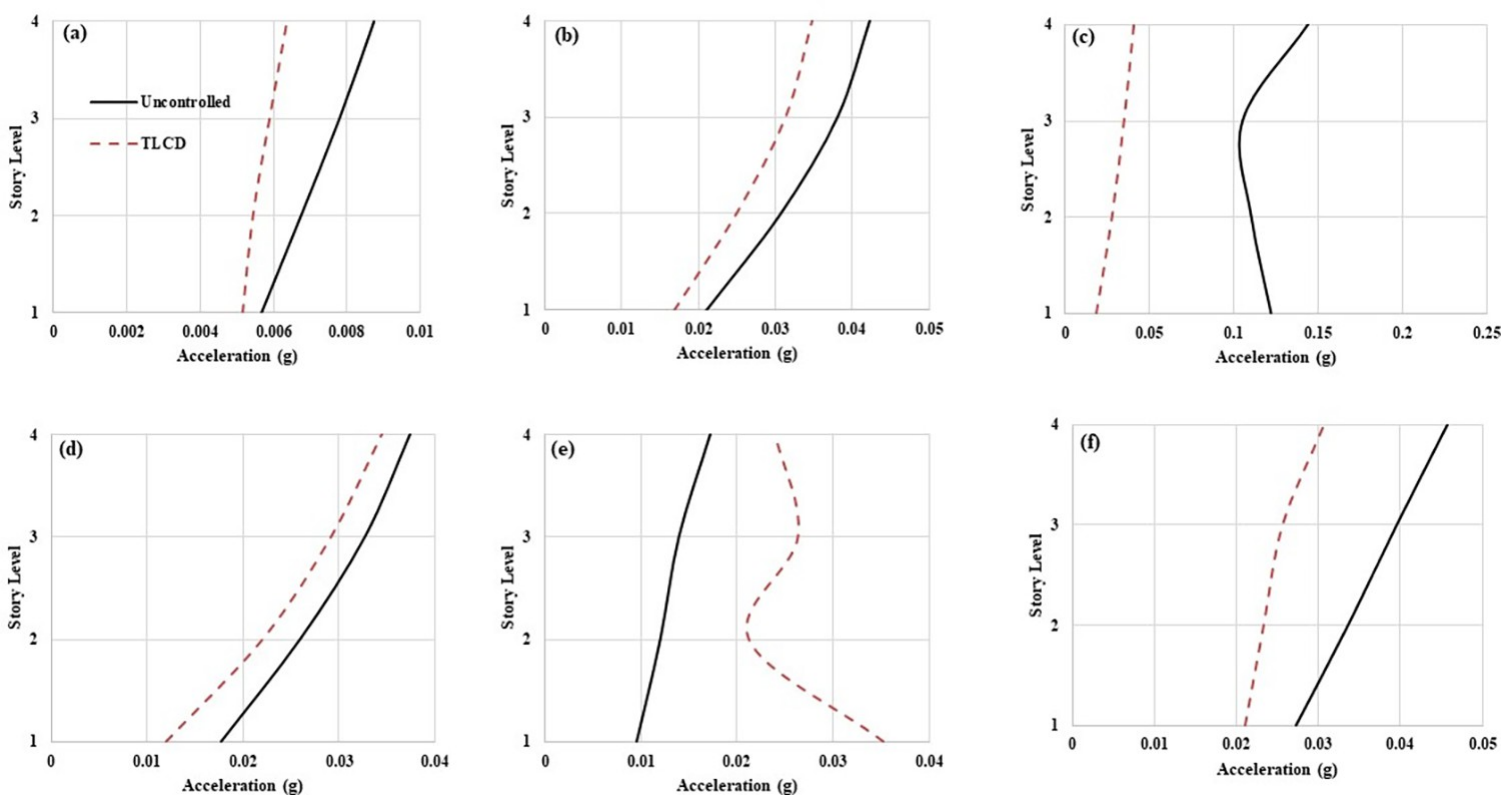
RMS acceleration responses of MDOF structure equipped with and without TLCD at (a) 0.65 Hz, (b) 1.17 Hz, (c) 1.3 Hz, (d) 1.43 Hz, (e) 1.95 Hz, and (f) Seismic loading.

The recorded acceleration data of all the loading types have been analyzed for the RMS and absolute peak acceleration values. Fig 7(A)–7(F) express the RMS accelerations responses of the structure at 0.65 Hz, 1.17 Hz, 1.30 Hz, 1.43 Hz, 1.95 Hz and seismic loadings respectively. It can be observed that controlled MDOF structure responses have been improved with TLCD at all loading conditions except 1.95 Hz. This slight increase in the response of the structure equipped with liquid dampers at loading frequencies higher than resonance loading frequency has been reported in the literature [15, 51]. It is because the liquid dampers are effective for low frequencies vibration and effective only in the resonance region. However, remarkable performance improvement has been observed in the MDOF structure with TLCD at the resonant point (1.30 Hz). In resonance, the uncontrolled structure RMS acceleration response at each storey level exceeds 0.1 g. On the other hand, MDOF equipped with TLCD has RMS accelerations values lower than 0.05 g at each degree of freedom. The slight increase in the response of the structure with TLCD at 1.95 Hz in comparison to the improvement that occurred at resonant loading with TLCD, is negligible and not problematic. Because at resonance the uncontrolled structure response reached up to 0.15 g which has been brought down to around 0.05 g with TLCD. The RMS acceleration response of the structure with TLCD has also been reduced at seismic loading compared to the uncontrolled system (Fig 7(F)).

**Fig 8:**
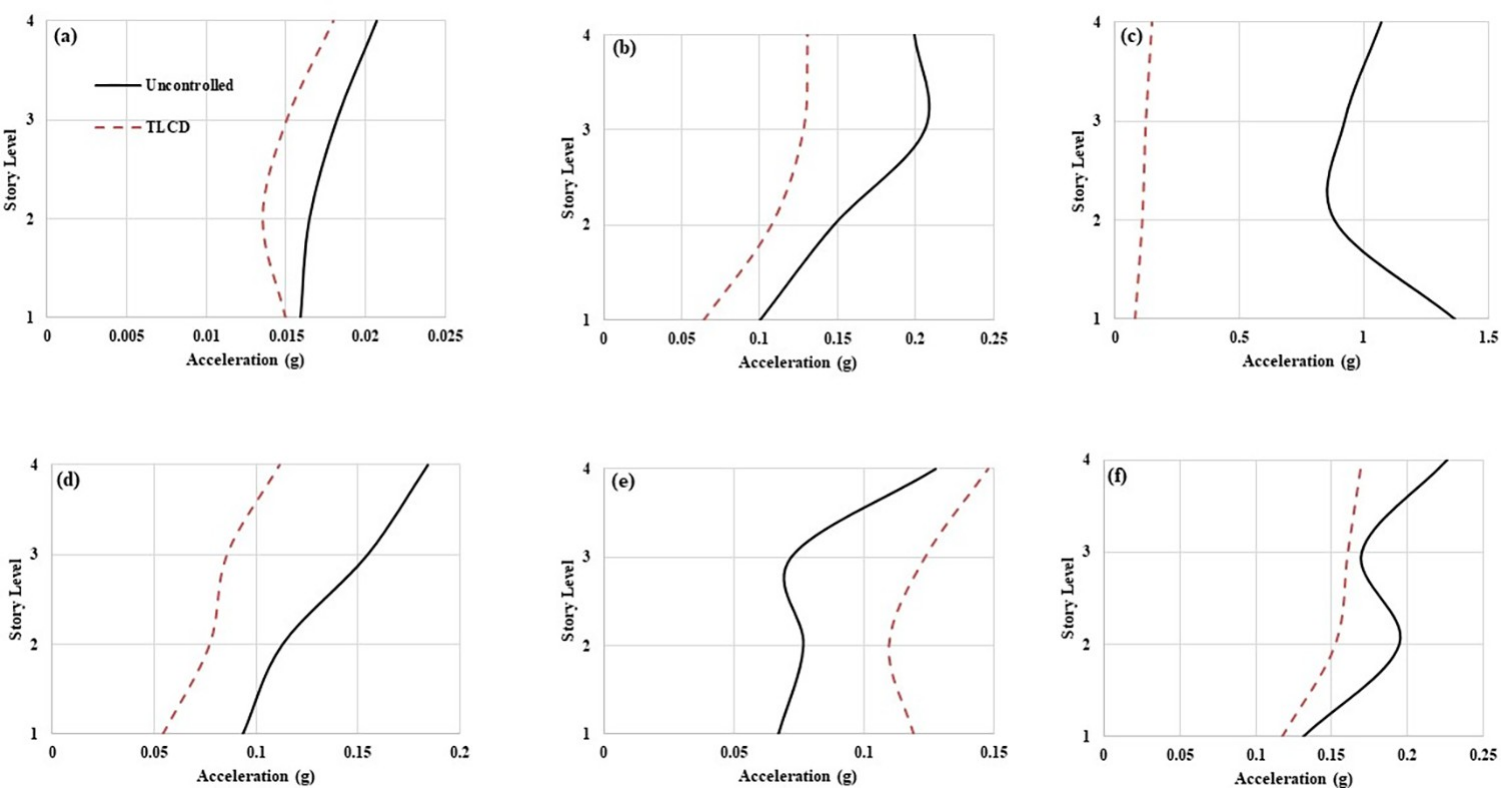
Peak acceleration responses of MDOF structure equipped with and without TLCD at (a) 0.65 Hz, (b) 1.17 Hz, (c) 1.3 Hz, (d) 1.43 Hz, (e) 1.95 Hz, and (f) Seismic loading.

Fig 8(A)–8(F) exhibit the absolute peak accelerations responses of MDOF structure at 0.65 Hz, 1.17 Hz, 1.30 Hz, 1.43 Hz, 1.95 Hz and EI Centro ground motion respectively. A similar trend to RMS accelerations responses can be observed in peak accelerations responses as well. Except for 1.95 Hz loading, at each storey level, the responses of the MDOF structure equipped with TLCD have been improved compared to the uncontrolled MDOF system. Exceptional improvement in the responses of structure with TLCD has occurred at 1.3 Hz loading (Wex/Ws = 1). At 1.3 Hz loading, the peak acceleration values from 1.36 g (Storey level-01) and 1.06 g (Storey level-04) have been reduced to 0.081g and 0.147 g respectively (Fig 6(C)). At storey level-04 the peak acceleration responses (Fig 6) have been decreased from 0.020 g (0.65 Hz), 0.19 g (1.17 Hz), 0.18g (1.43 Hz), and 0.22 g (EI Centro) to 0.017 g, 0.13 g, 0.11 g, and 0.16 g respectively. The MDOF structure equipped with TLCD has shown better performance in the mitigation of vibrations over uncontrolled structures due to the effective damping mechanism of TLCD. At 1.95 Hz (Wex/Ws = 1.5) loading at storey level-04 the MDOF structure peak acceleration value has been increased from 0.13 g to 0.14 g. But this increase in acceleration response of the structure with TLCD at 1.95 Hz is very low compared to improvements that have occurred with TLCD at resonant loading. Also, TLCDs are most effective and designed for controlling responses of the structure at critical loadings such as resonant loading (Wex/Ws = 1).

To get a better picture of TLCD performance to control structural vibrations, the percentage reductions in the RMS and peak accelerations have been computed. Table 4. demonstrates percentage reductions in the RMS and peak accelerations responses of MDOF structure with TLCD over uncontrolled MDOF structure at harmonic and seismic loadings. Where S-1, S-2, S-3 and S-4 denote first, second, third, and fourth storey levels respectively. Except for 1.95 Hz, at all other loading conditions both RMS and peak accelerations responses have been reduced. The maximum reduction in the RMS accelerations responses has been observed at 1.30 Hz loading for S-1, S-2, S-3, and S-4 are 85%, 75%, 67% and 72% respectively. At 1.3 Hz loading, the performance of TLCD in controlling structure responses is very prominent. The absolute peak accelerations of uncontrolled structure for S-1, S-2, S-3, and S-4 have been lowered to 94.06%, 87.56%, 86.60% and 86.18% with TLCD respectively. At seismic loading, the RMS accelerations responses of the structure with TLCD have been reduced to 22.72%, 30.57%, 35.12% and 32.74% for S-1, S-2, S-3, and S-4 respectively. While the maximum peak acceleration reduction has occurred for S-4 (24.81%) during earthquake loading. Among all types of loadings, the maximum reduction in the response of controlled structure at each storey level has occurred at 1.3 Hz loading.

**Table 4:** Percentage reduction in the responses of MDOF structure with TLCD over uncontrolled MDOF structure.

Loading Cases		S-1	S-2	S-3	S-4
	0.65 Hz	9.20	19.46	24.47	27.20
	1.17 Hz	20.01	19.20	18.01	18.00
	1.30 Hz	**85.18**	**75.15**	**67.11**	**71.88**
**RMS**	1.43 Hz	32.42	15.02	10.79	07.86
	1.95 Hz	-72.78	-43.31	-47.03	-28.14
	EI Centro	**22.72**	**30.57**	**35.12**	**32.74**
	0.65 Hz	05.81	17.85	17.23	12.93
	1.17 Hz	36.60	27.09	37.66	34.33
**Absolute Peak**	1.30 Hz	**94.06**	**87.56**	**86.60**	**86.18**
	1.43 Hz	42.27	31.67	44.65	39.44
	1.95 Hz	-43.57	-11.37	-33.56	-8.28
	EI Centro	10.05	21.50	5.22	**24.81**

### 4.3. RMS displacement and inter-storey drift response

**Fig 9:**
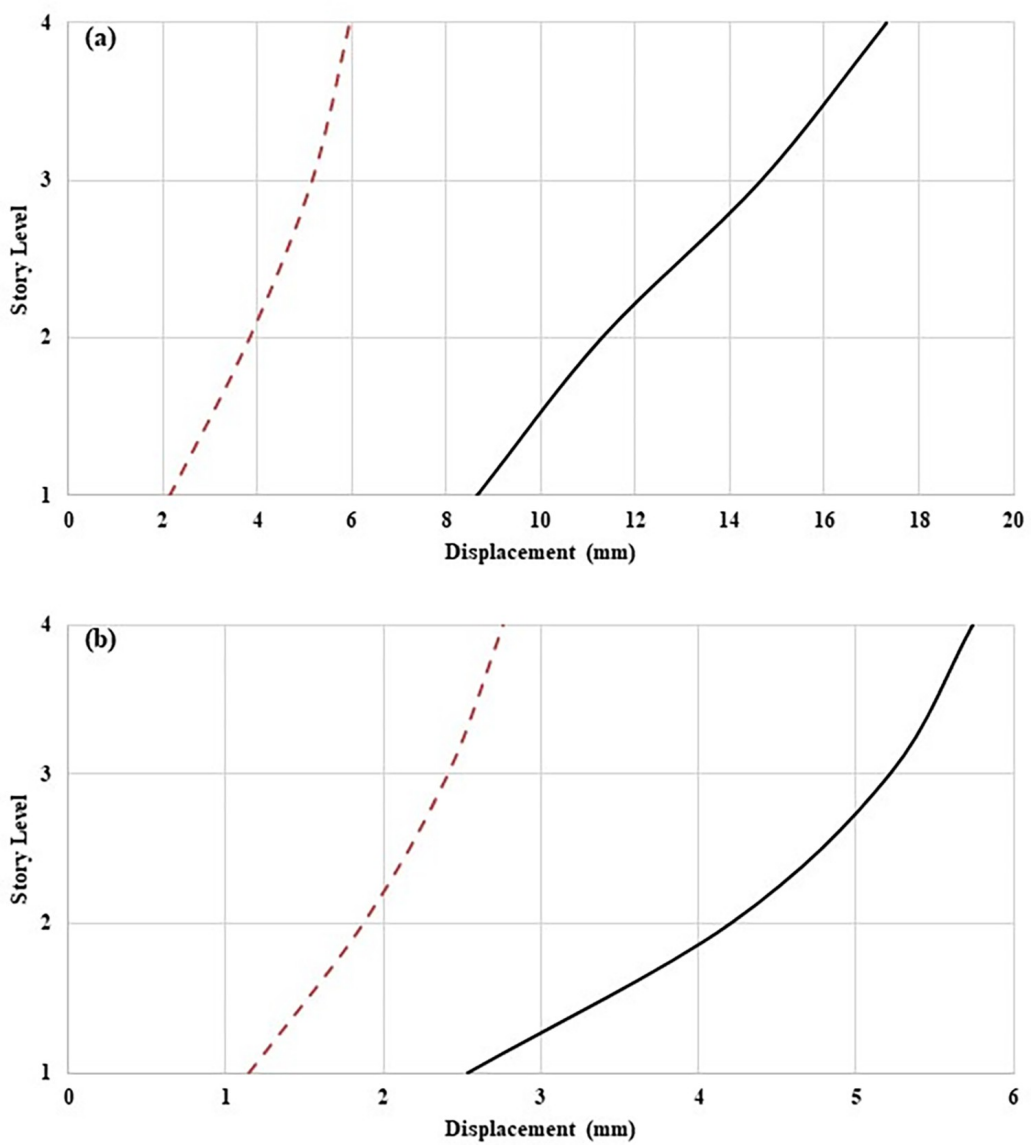
RMS displacement responses of MDOF structure equipped with and without TLCD at (a) Resonant loading, and (b) Seismic loading.

**Fig 10:**
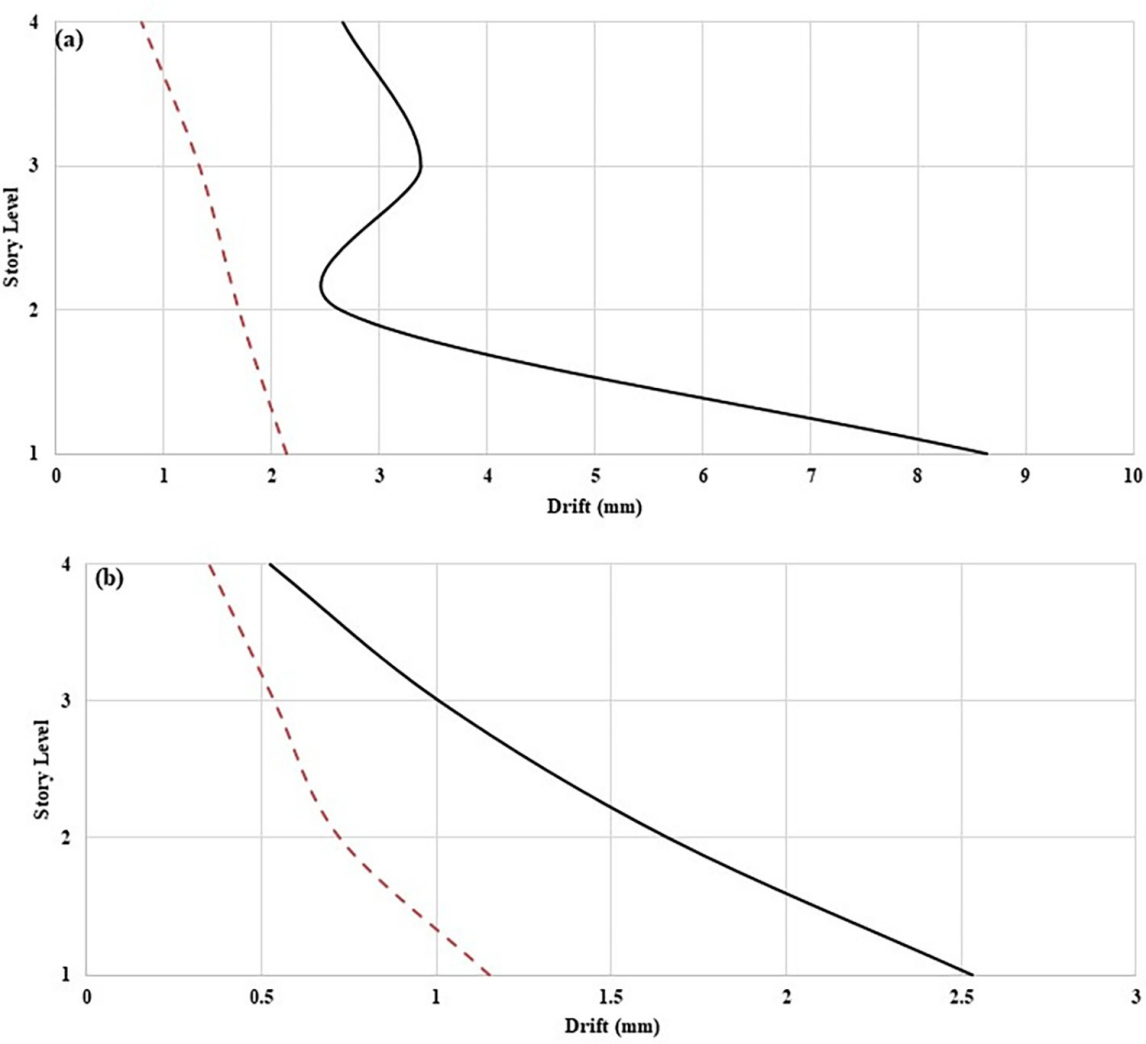
RMS inter-storey drifts responses of MDOF structure equipped with and without TLCD at (a) Resonant loading, and (b) Seismic loading.

**Fig 11:**
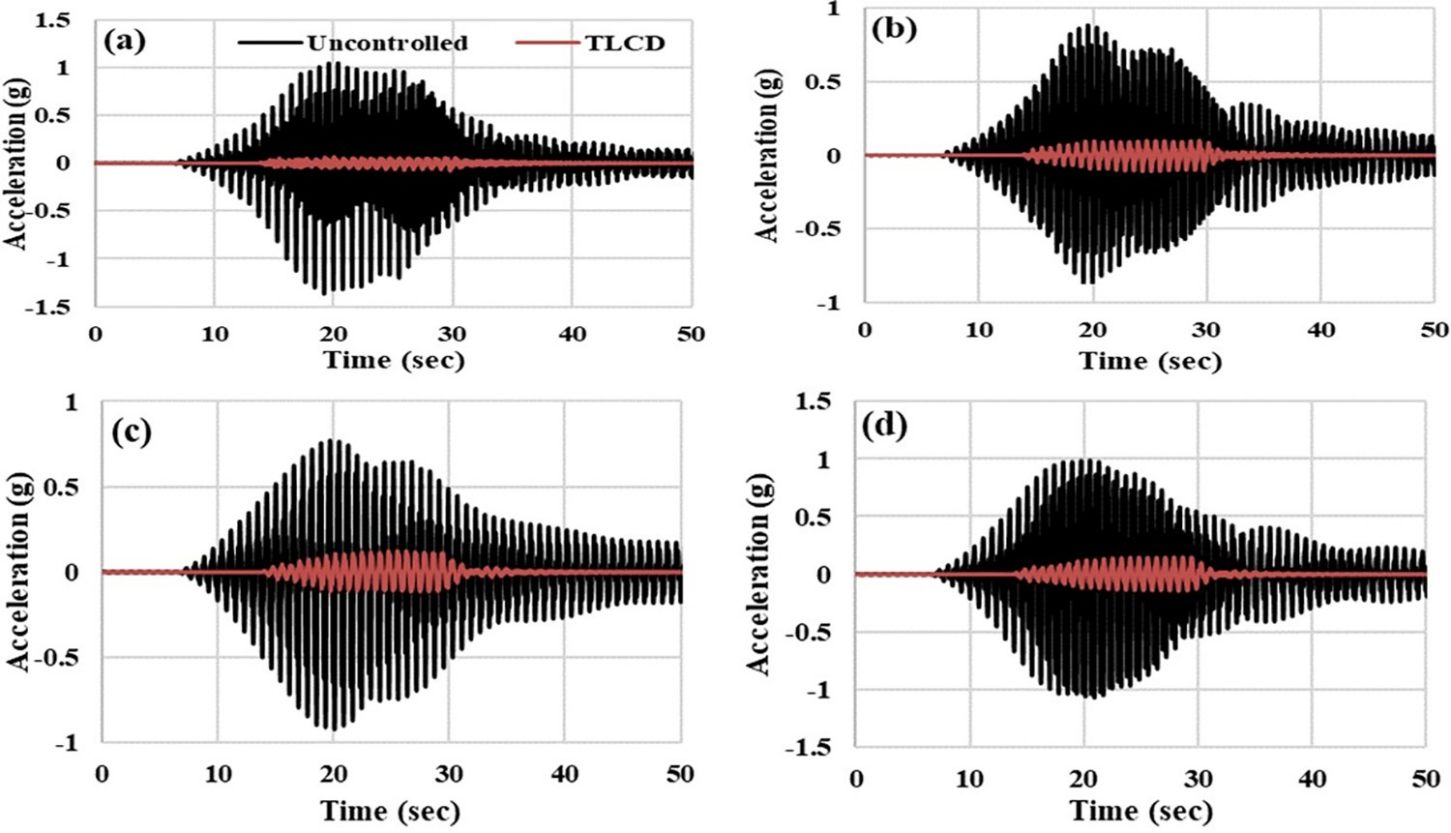
Acceleration time history responses of the MDOF structure equipped with and without TLCD at resonant loading, (a) Storey Level -01, (b) Storey Level -02, (c) Storey Level -03, (d) Storey Level -04.

The recorded acceleration data has been numerically integrated to determine the displacement responses of the structure for resonance and EL Centro seismic excitations. From the calculated displacement responses, RMS displacement and inter-storey drift have been determined. Fig 9(A) and 9(B) depict the RMS displacement responses under resonant and seismic excitations. TLCD reduced the displacement of the controlled structures at each storey level under both loadings conditions. The better external vibration mitigation of controlled structures is due to the damping mechanism of TLCD. A similar trend can be observed in the drift response. By equipping the structure with TLCD, the inter-storey drift has been reduced against resonant (Fig 10(A)) and seismic excitations (Fig 11(B)). Table 5 shows the percentage reduction in the displacement and drift responses of the controlled structure compared to the uncontrolled structure. In the case of resonance maximum improvement can be observed at storey level-01 in displacement and drift. While in the case of EL Centro ground motion maximum reduction in displacement and drift has occurred at storey level -02.

**Table 5:** Percentage reduction in the RMS displacement and drift responses of MDOF structure with TLCD over uncontrolled MDOF structure.

Loading Cases		S-1	S-2	S-3	S-4
**Displacement**	1.30 Hz	**75.22**	65.97	64.74	**65.55**
	EL Centro	**54.58**	**55.38**	53.77	51.93
**Drift**	1.30 Hz	**75.22**	35.71	60.64	**70.03**
	EL Centro	**54.58**	**56.61**	47.07	33.75

### 4.4. Analysis of acceleration response in time and frequency domains

**Fig 12:**
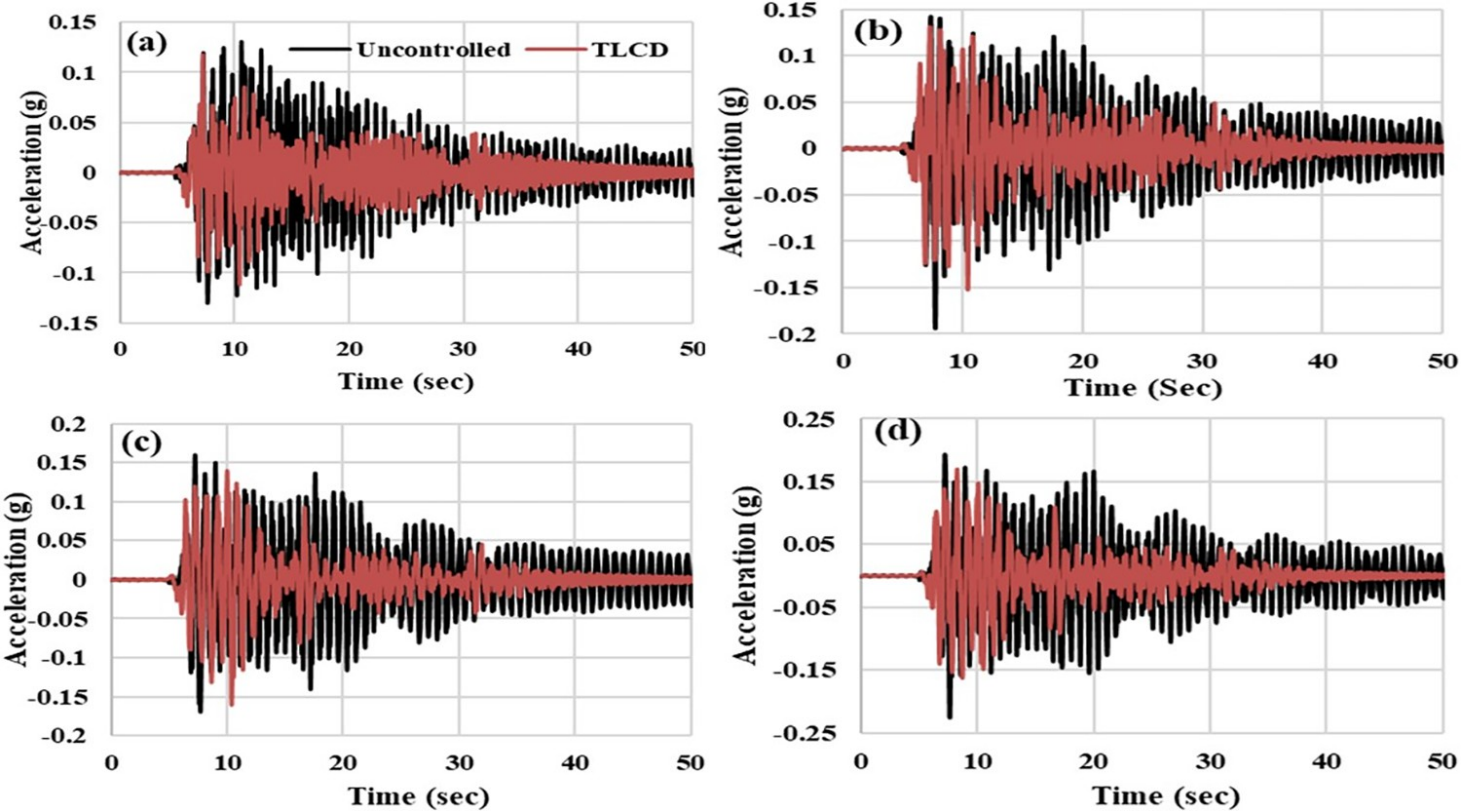
Acceleration time history responses of the MDOF structure equipped with and without TLCD at seismic loading, (a) Storey Level -01, (b) Storey Level -02, (c) Storey Level -03, (d) Storey Level -04.

To analyze the acceleration responses in the time domain, acceleration time histories of the MDOF structure with and without TLCD have been compared for harmonic and seismic loadings. Figs 11 and 12 show the time histories of MDOF structure at resonant (1.30 Hz) and seismic loadings respectively. In the case of harmonic loadings, the maximum improvement in the responses of MDOF structure with TLCD has been observed at the resonance point (Wex/Ws = 1). Therefore, time histories have been considered only for 1.3 Hz in the case of harmonic loading. Fig 11(A)–11(D) present the comparison of time histories of MDOF structure with and without TLCD at first, second, third and fourth storey levels respectively at 1.30 Hz. The TLCD has considerably reduced the accelerations responses of the structure at all four storeys. Acceleration peaks have been significantly reduced in MDOF structure responses equipped with TLCD compared to the uncontrolled system (Fig 11). Also, it is important to note that at resonant loading frequency, the structure has not achieved a completely steady-state condition because the number of cycles (20) has been kept constant for all harmonic loadings. Therefore, at 1.30 Hz loading due to a short interval of applied loading time, the state condition cannot be observed in time history responses (Fig 11). Under seismic loading, at first (Fig 12(A)), second (Fig 12(B)), third (Fig 12(C)), and fourth (Fig 12(D)) storey level the accelerations responses of MDOF structure with TLCD has been reduced compared to uncontrolled system. However, at harmonic loading (1.3 Hz), the mitigation of vibrational energies of MDOF structure with TLCD is very efficient and prominent compared to seismic loading. The improved performance of MDOF with TLCD compared to the uncontrolled system has been clearly reflected in time history responses.

**Fig 13:**
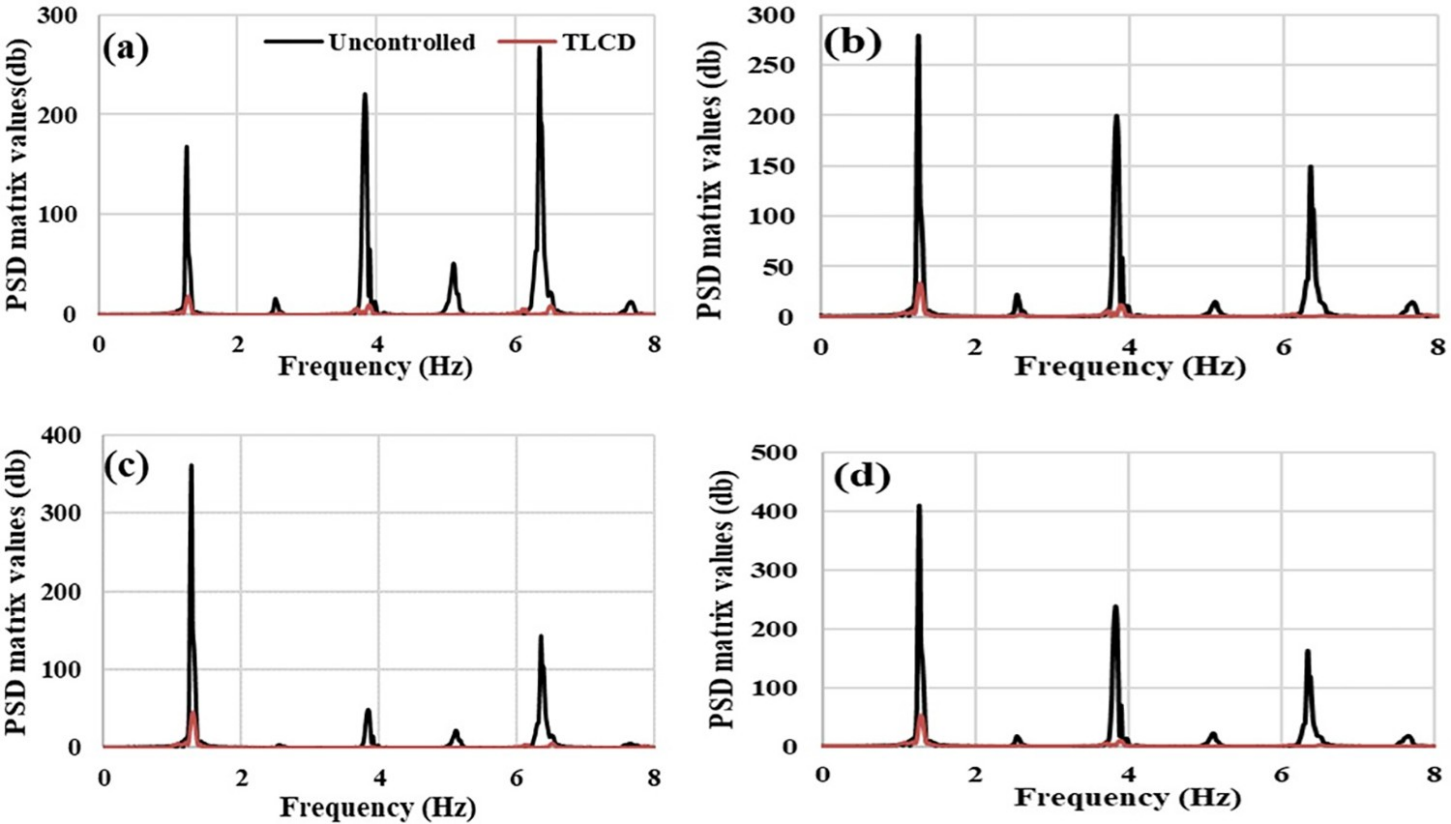
Acceleration power spectral density (PSD) responses of the MDOF structure equipped with and without TLCD at resonant loading, (a) Storey Level -01, (b) Storey Level -02, (c) Storey Level -03, (d) Storey Level -04.

To investigate the acceleration responses of the MDOF system in the frequency domain, the recorded acceleration data have been processed to find power spectral density (PSD) responses. Fast Fourier Transform (FFT) has been applied to acceleration data in the time domain to draw PSD curves. Fig 13(A)–13(D) represent the PSD curves of the controlled and uncontrolled system at the first, second, third, and fourth storey respectively at 1.3 Hz loading. Three dominant peaks can be observed in PSD responses of uncontrolled structure at each storey level. The first, second, and third peak have occurred at around 1.35 Hz, 3.70 Hz and 6.50 Hz respectively. These three peaks have been observed at frequencies that closely corresponded to the first (1.30 Hz), second (3.72 Hz) and fourth (6.82 Hz) vibrational modes frequencies of the MDOF structure. All these three peaks of uncontrolled MDOF structure have been suppressed in the MDOF system equipped with TLCD. The better performance of TLCD in controlling MDOF structure’s RMS and peak accelerations responses at resonant loading have been reflected in PSD curves in the form of suppression of dominant frequencies peaks.

**Fig 14:**
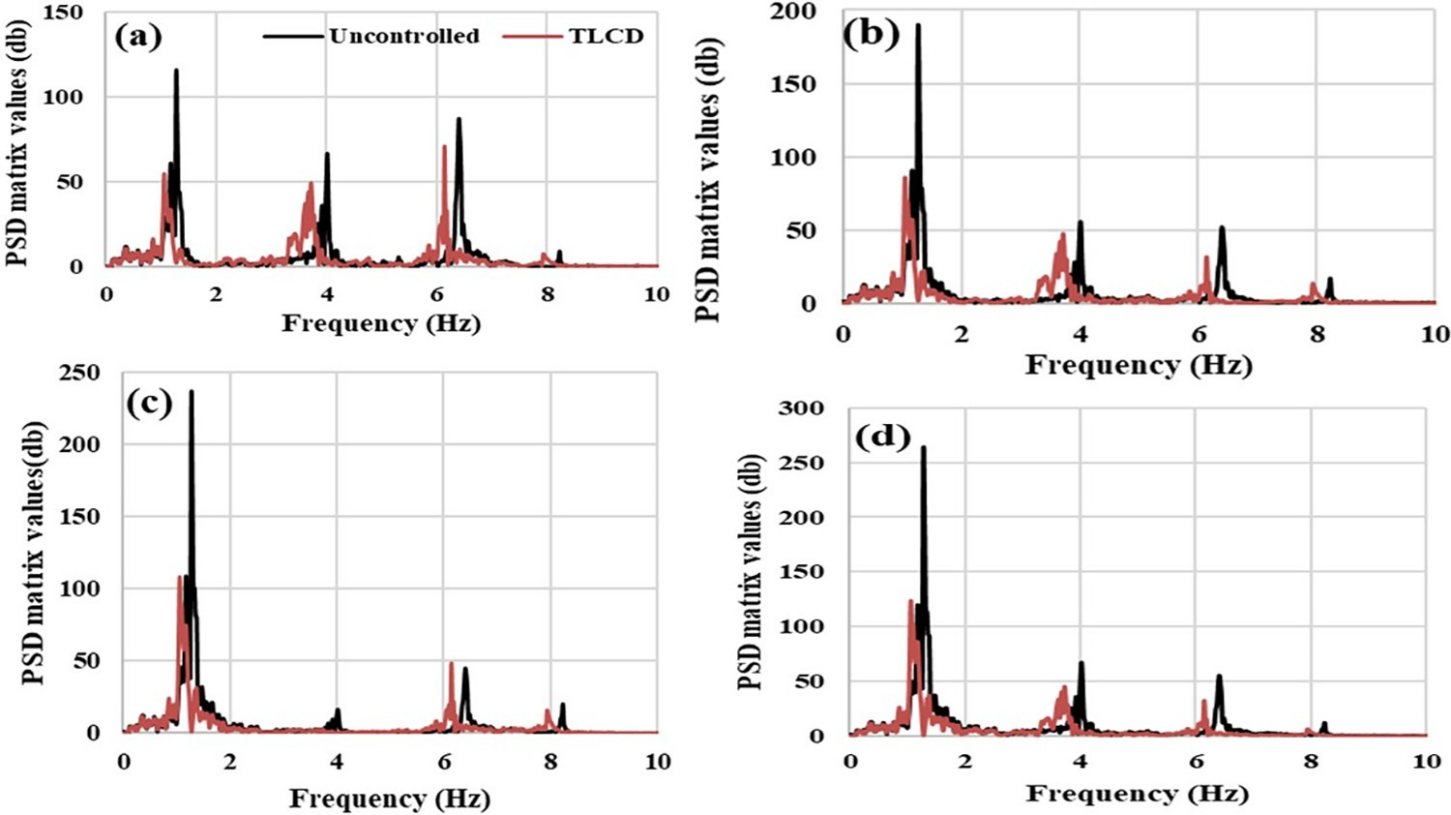
Acceleration Power Spectral Density (PSD) responses of the MDOF structure equipped with and without TLCD at seismic loading, (a) Storey Level -01, (b) Storey Level -02, (c) Storey Level -03, (d) Storey Level -04.

The PSD responses of the first, second, third, and fourth storey of MDOF systems are shown in Fig 14(A)–14(D) respectively for historic earthquake excitations. Three prominent PSD peaks can be observed in uncontrolled structure responses around 1.30 Hz, 4 Hz, and 6.40 Hz. These peaks have been subdued in MDOF structure with TLCD and shifted in the back direction. The suppression of these peaks is evidence of the improved performance of MDOF structure with TLCD over uncontrolled MDOF structure. A similar trend has been observed by analyzing the acceleration responses in the time domain.

## 5. Conclusions

This paper presents detailed experimental investigations on a four-storey frame structure equipped with TLCD. The effect of TLCD on the responses of the MDOF system has been studied under harmonic and seismic excitations. The major findings of the work are summarized below:

- The normalized frequency responses have been experimentally determined for both controlled and uncontrolled MDOF structures by applying a series of harmonic loadings. The responses of the MDOF structure equipped with TLCD have been improved at the loading frequency ratio (Wex/Ws) near the resonance point (0.9 to1.1) and before the resonant point (Wex/Ws< 0.9). At a loading frequency ratio (Wex/Ws) greater than 1.1 the TLCD slightly increased the response of the MDOF structure compared uncontrolled structure.
- At the resonance point (1.30 Hz) the responses of MDOF structure with TLCD have been significantly improved compared to the uncontrolled structure. RMS accelerations responses have been reduced to 85%, 75%, 67%, and 71% at the first, second, third and fourth storey levels of controlled structure respectively. The TLCD has decreased the peak acceleration responses of the controlled structure by more than 85% at each storey of the structure under 1.30 Hz loading.
- The TLCD has also reduced the RMS and peak acceleration responses of MDOF structure at historic earthquake ground motion excitations. The maximum reduction in RMS and peak accelerations have been noted at storey-03 (35%) and storey-04 (25%) respectively. However, at seismic loading in comparison to 1.30 Hz harmonic loading, improvement in the response of the MDOF system with TLCD is comparatively less. But still, the performance is better than the uncontrolled MDOF system.
- The TLCD has reduced the RMS displacement and drift responses of the MDOF structure under both seismic and resonant excitations. Thus equipping the structure with TLCD reduced the displacement and drift responses at each storey level because the external vibrational energies are efficiently damped by TLCD.
- The performance improvement in the responses of the structure with TLCD has been reflected in the frequency domain as well. Analyzing PSD curves at 1.30 Hz and seismic loadings it has been observed that MDOF equipped with TLCD has suppressed the dominant frequency peaks which is evidence of the improved performance of TLCD in controlling MDOF structure responses.

## References

[1] Halis Gunel M.; Emre Ilgin H. A proposal for the classification of structural systems of tall buildings. *Build*. *Environ*. 2007, 42, 2667–2675, doi: 10.1016/j.buildenv.2006.07.007

[2] Memon S.A.; Zain M.; Zhang D.; Rehman S.K.U.; Usman M.; Lee D. Emerging trends in the growth of structural systems for tall buildings. *J*. *Struct*. *Integr*. *Maint*. 2020, 5, 155–170, doi: 10.1080/24705314.2020.1765270

[3] Jafari M.; Alipour A. Methodologies to mitigate wind-induced vibration of tall buildings: A state-of-the-art review. *J*. *Build*. *Eng*. 2021, 33, 101582, doi: 10.1016/j.jobe.2020.101582

[4] Kwok K.C.S.; Hitchcock P.A.; Burton M.D. Perception of vibration and occupant comfort in wind-excited tall buildings. *J*. *Wind Eng*. *Ind*. *Aerodyn*. 2009, 97, 368–380, doi: 10.1016/j.jweia.2009.05.006

[5] Baskaran A. Wind engineering studies on tall buildings-transitions in research. *Build*. *Environ*. 1993, 28, 1–19, doi: 10.1016/0360-1323(93)90002-K

[6] Di Matteo A.; Pirrotta A.; Tumminelli S. Combining TMD and TLCD: analytical and experimental studies. *J*. *Wind Eng*. *Ind*. *Aerodyn*. 2017, 167, 101–113, doi: 10.1016/j.jweia.2017.04.010

[7] Zain M.; Usman M.; Farooq S.H.; Mehmood T. Seismic Vulnerability Assessment of School Buildings in Seismic Zone 4 of Pakistan. *Adv*. *Civ*. *Eng*. 2019, 2019, doi: 10.1155/2019/5808256

[8] Tanveer M.; Usman M.; Khan I.U.; Farooq S.H.; Hanif A. Material optimization of tuned liquid column ball damper (TLCBD) for the vibration control of multi-storey structure using various liquid and ball densities. *J*. *Build*. *Eng*. 2020, 32, 101742, doi: 10.1016/j.jobe.2020.101742

[9] Khan B.L.; Farooq H.; Usman M.; Butt F.; Khan A.Q.; Hanif A. Effect of soil–structure interaction on a masonry structure under train-induced vibrations. *Proc*. *Inst*. *Civ*. *Eng*.*—Struct*. *Build*. 2019, 172, 1–13, doi: 10.1680/jstbu.18.00131

[10] Hassan M.A.; Usman M.; Hanif A.; Farooq S.H.; Ahmed J. Improving structural performance of timber wall panels by inexpensive FRP retrofitting techniques. *J*. *Build*. *Eng*. 2020, 27, 101004, doi: 10.1016/j.jobe.2019.101004

[11] Farooq H.; Usman M.; Mehmood K.; Malik M.S.; Hanif A. Effect of Steel Confinement on Axially Loaded Short Concrete Columns. *IOP Conf*. *Ser*. *Mater*. *Sci*. *Eng*. 2018, 414, doi: 10.1088/1757-899X/414/1/012026

[12] Khan R.; Farooq S.H.; Usman M. Blast Loading Response of Reinforced Concrete Panels Externally Reinforced with Steel Strips. *Infrastructures* 2019, 4, 54, doi: 10.3390/infrastructures4030054

[13] Ahmad J.; Usman M.; Hassan M.A.; Farooq S.H.; Hanif A. Enhancing Lateral Load Performance of Traditional Timber Wall (Dhajji-Dewari) by Strengthening of joints. *IOP Conf*. *Ser*. *Mater*. *Sci*. *Eng*. 2018, 431, doi: 10.1088/1757-899X/431/7/072002

[14] Hafeez M.A.; Usman M.; Umer M.A.; Hanif A. Recent Progress in Isotropic Magnetorheological Elastomers and Their Properties: A Review. 2020, 12, 1–37. 10.3390/polym12123023 PMC7766993 33348727

[15] Shah M.U.; Usman M.; Farooq S.H.; Kim I.-H. Effect of Tuned Spring on Vibration Control Performance of Modified Liquid Column Ball Damper. *Appl*. *Sci*. 2021, 12, 318, doi: 10.3390/app12010318

[16] Khan B.L.; Azeem M.; Usman M.; Farooq S.H.; Hanif A.; Fawad M. Effect of near and far Field Earthquakes on performance of various base isolation systems. *Procedia Struct*. *Integr*. 2019, 18, 108–118, doi: 10.1016/j.prostr.2019.08.145

[17] Khayam S.U.; Usman M.; Umer M.A.; Rafique A. Development and characterization of a novel hybrid magnetorheological elastomer incorporating micro and nano size iron fillers. *Mater*. *Des*. 2020, 192, doi: 10.1016/j.matdes.2020.108748

[18] Khan I.U.; Usman M.; Tanveer M. Vibration control of an irregular structure using single and multiple tuned mass dampers. *Proc*. *Inst*. *Civ*. *Eng*.*—Struct*. *Build*. 2021, 12, 1–26, doi: 10.1680/jstbu.21.00011

[19] Ghaedi K.; Ibrahim Z.; Adeli H.; Javanmardi A. Invited review: Recent developments in vibration control of building and bridge structures. *J*. *Vibroengineering* 2017, 19, 3564–3580, doi: 10.21595/jve.2017.18900

[20] Shah M.U.; Usman M. Feasibility Study of Liquid Tuned Column Hollow Ball Damper for Vibration Control of Structures. 2021, 1–13.

[21] Min L. Studies on Tuned Liquid Damper (Tld) By Free-Oscillation. 1988, 5, 381–391.

[22] Balendra T.; Wang C.M.; Cheong H.F. Effectiveness of tuned liquid column dampers for vibration control of towers. *Eng*. *Struct*. 1995, 17, 668–675, doi: 10.1016/0141-0296(95)00036-7

[23] Fujiv K. Suppression of Wind-Induced Vibration of a Tall Building using Tuned Liquid. 1992, 4, 1895–1906.

[24] Tamura Y.; Fujii K.; Ohtsuki T.; Wakahara T.; Kohsaka R. Effectiveness of tuned liquid dampers under wind excitation. *Eng*. *Struct*. 1995, 17, 609–621, doi: 10.1016/0141-0296(95)00031-2

[25] Chang C.C.; Gu M. Suppression of vortex-excited vibration of tall buildings using tuned liquid dampers. *J*. *Wind Eng*. *Ind*. *Aerodyn*. 1999, 83, 225–237, doi: 10.1016/S0167-6105(99)00074-4

[26] Banerji P.; Murudi M.; Shah A.H.; Popplewell N. Tuned liquid dampers for controlling earthquake response of structures. *Earthq*. *Eng*. *Struct*. *Dyn*. 2000, 29, 587–602, doi: 10.1002/(SICI)1096-9845(200005)29:5&lt;587::AID-EQE926&gt;3.0.CO;2-I

[27] Pandey D.K.; Sharma M.K.; Mishra S.K. A compliant tuned liquid damper for controlling seismic vibration of short period structures. *Mech*. *Syst*. *Signal Process*. 2019, 132, 405–428, doi: 10.1016/j.ymssp.2019.07.002

[28] Zhang Z.; Staino A.; Basu B.; Nielsen S.R.K. Performance evaluation of full-scale tuned liquid dampers (TLDs) for vibration control of large wind turbines using real-time hybrid testing. *Eng*. *Struct*. 2016, 126, 417–431, doi: 10.1016/j.engstruct.2016.07.008

[29] Tait M.J.; Isyumov N.; El Damatty A.A. Effectiveness of a 2D TLD and Its Numerical Modeling. *J*. *Struct*. *Eng*. 2007, 133, 251–263, doi: 10.1061/(asce)0733-9445(2007)133:2(251)

[30] Love J.S.; Tait M.J. Nonlinear simulation of a tuned liquid damper with damping screens using a modal expansion technique. *J*. *Fluids Struct*. 2010, 26, 1058–1077, doi: 10.1016/j.jfluidstructs.2010.07.004

[31] SAKAI F. Tuned liquid column damper-new type device for suppression of building vibration. In Proceedings of the Proceedings of 1^<st> International Conference on High-rise Buildings; Beijing,China, 1989; pp. 926–931.

[32] Chang C.C.; Hsu C.T. Control performance of liquid column vibration absorbers. *Eng*. *Struct*. 1998, 20, 580–586, doi: 10.1016/S0141-0296(97)00062-X

[33] Wang Q.; Tiwari N.D.; Qiao H.; Wang Q. Inerter-based tuned liquid column damper for seismic vibration control of a single-degree-of-freedom structure. *Int*. *J*. *Mech*. *Sci*. 2020, 184, doi: 10.1016/j.ijmecsci.2020.105840

[34] Alkmim M.H.; Fabro A.T.; de Morais M.V.G. Optimization of a tuned liquid column damper subject to an arbitrary stochastic wind. *J*. *Brazilian Soc*. *Mech*. *Sci*. *Eng*. 2018, 40, 1–11, doi: 10.1007/s40430-018-1471-3

[35] Xu Y.L.; Samali B.; Kwok K.C.S. Control of Along‐Wind Response of Structures by Mass and Liquid Dampers. *J*. *Eng*. *Mech*. 1992, 118, 20–39, doi: 10.1061/(ASCE)0733-9399(1992)118:1(20)

[36] Koh C.G.; Mahatma S.; Wang C.M. Theoretical and experimental studies on rectangular liquid dampers under arbitrary excitations. *Earthq*. *Eng*. *& Struct*. *Dyn*. 1994, 23, 17–31, doi: 10.1002/eqe.4290230103

[37] Sun L.M.; Fujino Y.; Koga K. A model of tuned liquid damper for suppressing pitching motions of structures. *Earthq*. *Eng*. *& Struct*. *Dyn*. 1995, 24, 625–636, doi: 10.1002/eqe.4290240502

[38] Xue S.D.; Ko J.M.; Xu Y.L. Optimum parameters of tuned liquid column damper for suppressing pitching vibration of an undamped structure. *J*. *Sound Vib*. 2000, 235, 639–653, doi: 10.1006/jsvi.2000.2947

[39] Shum K.M. Closed form optimal solution of a tuned liquid column damper for suppressing harmonic vibration of structures. *Eng*. *Struct*. 2009, 31, 84–92, doi: 10.1016/j.engstruct.2008.07.015

[40] Wu J.C.; Shih M.H.; Lin Y.Y.; Shen Y.C. Design guidelines for tuned liquid column damper for structures responding to wind. *Eng*. *Struct*. 2005, 27, 1893–1905, doi: 10.1016/j.engstruct.2005.05.009

[41] Colwell S.; Basu B. Investigations on the performance of a liquid column damper (LCD) with different orifice diameter ratios. *Can*. *J*. *Civ*. *Eng*. 2011, 33, 588–595, doi: 10.1139/l06-016

[42] Das S.; Choudhury S. Seismic response control by tuned liquid dampers for low-rise RC frame buildings. *Aust*. *J*. *Struct*. *Eng*. 2017, 18, 135–145, doi: 10.1080/13287982.2017.1351180

[43] Xin Y.; Chen G.; Lou M. Seismic response control with density-variable tuned liquid dampers. *Earthq*. *Eng*. *Eng*. *Vib*. 2009, 8, 537–546, doi: 10.1007/s11803-009-9111-7

[44] Gao H.; Kwok K.C.S.; Samali B. Optimization of tuned liquid column dampers. *Eng*. *Struct*. 1997, 19, 476–486, doi: 10.1016/S0141-0296(96)00099-5

[45] Hitchcock P.A.; Kwok K.C.S.; Watkins R.D.; Samali B. Characteristics of liquid column vibration absorbers (LCVA)—I. *Eng*. *Struct*. 1997, 19, 126–134, doi: 10.1016/S0141-0296(96)00042-9

[46] Hitchcock P.A.; Kwok K.C.S.; Watkins R.D.; Samali B. Characteristics of liquid column vibration absorbers (LCVA)—II. *Eng*. *Struct*. 1997, 19, 135–144, doi: 10.1016/S0141-0296(96)00044-2

[47] Ghosh A.; Basu B. Seismic vibration control doi: 10.1016/j.engstruct.2004.07.001

[48] Huo L.; Li H. 13 th World Conference onof short period structures using the liquid column damper. Eng. Struct. 2004, 26, 1905–1913, d Earthquake Engineering TORSIONALLY COUPLED RESPONSE CONTROL OF STRUCTURES. In Proceedings of the 13 th World Conference on Earthquake Engineering; 2004.

[49] Mehrkian B.; Altay O. Mathematical modeling and optimization scheme for omnidirectional tuned liquid column dampers. *J*. *Sound Vib*. 2020, 484, doi: 10.1016/j.jsv.2020.115523

[50] Ding H.; Wang J.T.; Lu L.Q.; Zhu F. A toroidal tuned liquid column damper for multidirectional ground motion-induced vibration control. *Struct*. *Control Heal*. *Monit*. 2020, 27, 1–19, doi: 10.1002/stc.2558

[51] Al-Saif K.A.; Aldakkan K.A.; Foda M.A. Modified liquid column damper for vibration control of structures. *Int*. *J*. *Mech*. *Sci*. 2011, 53, 505–512, doi: 10.1016/j.ijmecsci.2011.04.007

[52] Shum K.M.; Xu Y.L. Multiple tuned liquid column dampers for reducing coupled lateral and torsional vibration of structures. *Eng*. *Struct*. 2004, 26, 745–758, doi: 10.1016/j.engstruct.2004.01.006

[53] Shum K.M.; Xu Y.L.; Guo W.H. Wind-induced vibration control of long span cable-stayed bridges using multiple pressurized tuned liquid column dampers. *J*. *Wind Eng*. *Ind*. *Aerodyn*. 2008, 96, 166–192, doi: 10.1016/j.jweia.2007.03.008

[54] Gur S.; Roy K.; Mishra S.K. Tuned liquid column ball damper for seismic vibration control. *Struct*. *Control Heal*. *Monit*. 2015, 22, 1325–1342, doi: 10.1002/stc.1740

[55] Min K.W.; Kim H.S.; Lee S.H.; Kim H.; Kyung Ahn S. Performance evaluation of tuned liquid column dampers for response control of a 76-story benchmark building. *Eng*. *Struct*. 2005, 27, 1101–1112, doi: 10.1016/j.engstruct.2005.02.008

[56] Kuriakose Riju; Lakshmi P. Effectiveness of Tuned Liquid Dampers on High Rise Buildings in Kerala. *Int*. *J*. *Eng*. *Res*. 2016, V5, 24–30, doi: 10.17577/ijertv5is090056

[57] Zhu F.; Wang J.T.; Jin F.; Lu L.Q. Real-time hybrid simulation of full-scale tuned liquid column dampers to control multi-order modal responses of structures. *Eng*. *Struct*. 2017, 138, 74–90, doi: 10.1016/j.engstruct.2017.02.004

[58] Structures M.; Ocak A.; Bekda G. Optimization of Tuned Liquid Damper Including Different. 2022.

[59] Tanveer M.; Usman M.; Khan I.U.; Ahmad S.; Hanif A.; Farooq S.H. Application of tuned liquid column ball damper (TLCBD) for improved vibration control performance of multi-storey structure. *PLoS One* 2019, 14, 1–15, doi: 10.1371/journal.pone.0224436 31648266 PMC6812827

[60] Inamdar N.J. *Educational shaking table modules for earthquake engineering*; 2010.

